# 1241. Outcomes of a Novel Antimicrobial Handshake Stewardship Service in the Immunocompromised Population at an Academic Medical Center

**DOI:** 10.1093/ofid/ofad500.1081

**Published:** 2023-11-27

**Authors:** Keven Gomez, Richelle Guerrero-Wooley, Caleb C McLeod, Abdulwhab Shremo Msdi, Sandy Chang, Jonathan T Arcobello, Anna Zhou

**Affiliations:** University of California San Diego Health, San Diego, California; Loma Linda University Heatlh, Loma Linda, California; Santa Clara Valley Medical Center, San Jose , California; Loma linda, Loma linda, California; Loma Linda University, Loma Linda, California; Loma Linda University Medical Center, Loma Linda, California; Loma Linda University, Loma Linda, California

## Abstract

**Background:**

Prospective audit and feedback (PAF) is integral to antimicrobial stewardship programs (ASP) and is typically conducted electronically. Handshake stewardship (HS) is a form of in-person PAF advocated by the CDC and IDSA. There is scarce data regarding successful ASP interventions within the immunocompromised (IC) population, and even less data on the impact of HS. The objective of this study was to describe the outcomes of a novel HS service on the IC population and identify ASP interventions that may be generalizable.

**Methods:**

This is a retrospective study of hospitalized adult patients (≥ 18 years old) with IC conditions on pre-determined antimicrobials for at least 72 hours between May 1^st^, 2022 and January 31^st^, 2023. IC patients included those with active malignancy on chemotherapy or with functional immunosuppression, solid organ transplant (SOT), and rheumatological conditions on active immunosuppressants. The ASP team comprised of 1 infectious disease (ID) physician and 1 ID pharmacist who provided all recommendations to the intensive care unit (ICU), transplant, and oncology services. Primary outcomes included recommendation acceptance and recommendation types. Additional outcomes included incidence of *C. difficile*, resumption of antibiotics after discontinuation, lengths of stay (LOS), and 60-day readmission and mortality. Descriptive statistics were performed.

**Results:**

Twenty-nine patients with IC conditions were identified. Majority were White (41.4%) or Hispanic/Latino (44.8%). The median age was 57 years old (IQR 22-78). Fifteen patients had active malignancy, 11 were SOT recipients, and 3 had rheumatologic disorders. Primary teams accepted 45/51 (85%) of recommendations for 27/29 (93.1%) patients. Common recommendations included discontinuation of empiric therapy for febrile neutropenia or for non-infectious etiologies, de-escalation, or escalation to ID consults. Primary teams only resumed antibiotics in 1 patient after early discontinuation. The median LOS was 14 days with 2 patients testing positive for *C. difficile* and only 1 patient readmitted due to infection.

**Conclusion:**

HS was well received and most recommendations targeting the IC population were accepted and did not lead to poor patient outcomes.

**Disclosures:**

**Anna Zhou, PharmD, BCIDP**, Melinta Therapeutics: Honoraria

